# Pre-weaning socialisation with non-littermates in piglets: reduced weaning stress when regrouped with unfamiliar piglets post-weaning?

**DOI:** 10.18849/ve.v8i3.664

**Published:** 2023-09-27

**Authors:** Dongyue Du+, Jenny-Ann Toribio+

**Keywords:** PRE-WEANING SOCIALISATION, PIGLET, WEANING STRESS, POST-WEANING REGROUPING

## Abstract

**PICO question:**

In piglets in indoor housing systems does pre-weaning socialisation with non-littermates compared to no pre-weaning socialisation with non-littermates result in lower weaning stress when regrouped with unfamiliar piglets post-weaning?

**Clinical bottom line:**

**Category of research:**

Treatment.

**Number and type of study designs reviewed:**

Nine papers were critically reviewed. All of them were controlled trials, of which two were randomised control trials.

**Strength of evidence:**

Moderate.

**Outcomes reported:**

In terms of behavioural indicators of weaning stress, piglets socialised with non-littermates during lactation showed less aggressive behaviours (in the six papers that investigated aggression) and fewer skin lesions (in the six out of seven papers that investigated skin lesions) than non-socialised piglets when regrouped with unfamiliar piglets post-weaning. One of two papers that measured cortisol levels in piglets showed that the cortisol level of socialised piglets had a smaller increase from pre-weaning to post-weaning level than non-socialised piglets. Four out of five papers that investigated average daily weight gain (ADWG) found no significant difference between socialised and non-socialised piglets.

**Conclusion:**

Based on behavioural indicators, there was strong evidence suggesting that pre-weaning socialisation with non-littermates could reduce aggression and skin lesions when regrouped with unfamiliar piglets post-weaning. However, due to the weak evidence for the physiological indicator and growth performance, the effect of pre-weaning socialisation on weaning stress was inconclusive.

## How to apply this evidence in practice

The application of evidence into practice should take into account multiple factors, not limited to: individual clinical expertise, patient’s circumstances and owners’ values, country, location or clinic where you work, the individual case in front of you, the availability of therapies and resources.

Knowledge Summaries are a resource to help reinforce or inform decision making. They do not override the responsibility or judgement of the practitioner to do what is best for the animal in their care.

## Clinical scenario

Weaning is a stressful event for piglets in indoor commercial piggeries. Piglets are challenged by multiple stressors simultaneously, such as separation from sow and littermates, the transition from milk to a solid diet, and the introduction to a new housing environment (Campbell et al., 2013). As a result, reduced feed intake, stagnation of growth, and changes in behaviour such as increased aggression, are expected to occur during the two weeks following weaning (Dybkjaer, 1992; Campbell et al., 2013). Weaning stress also results in intestinal barrier function disruption and increased vulnerability to pathogens, which leads to malabsorption and diarrhoea, exacerbating the production loss (Campbell et al., 2013).

Regrouping piglets from two or more litters at weaning to form weaner groups of 20 or more piglets is standard practice to align group size with the space and feeding facility of the grower pig accommodation (O'Connell et al., 2004). Reconstituted groups are more homogenised in piglet weight, resulting in a more uniform weight at slaughter (O'Connell et al., 2005). Despite these advantages, regrouping contributes significantly to social stress in piglets. Piglets placed into a new group are required to re-establish a dominant social hierarchy through fighting (van Putten & Buré, 1997). As such, the social skills or ‘fighting strategy’ of piglets are critical, which include appropriate recognition of the threat, initiation of the fight, prediction of fighting ability, and timely withdrawal from the fight by showing submissive behaviours (van Putten & Buré, 1997). These skills prevent piglets from engaging in long and unnecessary fights and help piglets establish new social hierarchies quickly (van Putten & Buré, 1997). In nature, suckling piglets co-mingle with piglets from other litters and gradually build up such social skills (Jensen, 1986); however, in indoor systems that utilise farrowing crates, pre-weaning interaction with non-littermates is non-existent. Though some indoor housing arrangements, such as group housing, present opportunities for social mixing, they are relatively unpopular in commercial piggeries due to various concerns, such as higher pre-weaning piglet mortality and increased cost associated with larger pen area (Baxter et al., 2012).

Early socialisation of the piglets is an approach to enable interaction with unfamiliar piglets during lactation through special housing designs, in which piglets can learn social skills that are applied to the re-establishment of social hierarchy after post-weaning regrouping (Blavi et al., 2021). Early socialisation of piglets usually commences from Day 7–14 post-parturition until weaning (Salazar et al., 2018). Socialisation can be facilitated by housing designs such as a passage between two neighbouring farrowing crates (Salazar et al., 2018), accessible common areas for piglets in 3–5 litters (Weary et al., 1999), or a group lactation pen (van Nieuwamerongen et al., 2015). It is hypothesised that piglets with early socialisation will be more confident when they are mixed post-weaning, smoothing the transition, reducing aggression, and consequently improving post-weaning growth performance (Blavi et al., 2021). However, producers may be reluctant to adopt early socialisation practices as the extent, nature and significance of positive impact require further delineation and justification (Baxter et al., 2012).

This study aimed to review the evidence on the impact of pre-weaning socialisation with non-littermates on piglet weaning stress when regrouped with unfamiliar piglets post-weaning.

## The evidence

Nine papers were critically appraised. All were controlled trials, of which two were randomised control trials (Ji et al., 2021; Salazar et al., 2018). The outcomes measured for weaning stress were categorised into physiological, behavioural, and growth performance. In the physiological category, two papers measured cortisol level (Ji et al., 2021; and Salazar et al., 2018). In the behavioural category, six papers measured aggressive behaviours (D’Eath, 2005; Fels et al., 2021; Ji et al., 2021; Kanaan et al., 2012; Verdon et al., 2016; and Verdon et al., 2019), and seven papers measured skin lesions (Camerlink et al., 2018; D’Eath, 2005; Fels et al., 2021; Kanaan et al., 2012; Salazar et al., 2018; Schrey et al., 2019; and Verdon et al., 2016). In the growth performance category, five papers measured average daily weight gain (ADWG) (D’Eath, 2005; Ji et al., 2021; Kanaan et al., 2012; Salazar et al., 2018; and Schrey et al., 2019). Comparisons between papers were challenging due to highly variable study designs in socialisation housing design, socialisation length, weaning age, post-weaning group size, and methods of outcome measurement.

Overall, there was strong evidence (in the six papers that investigated aggression) that pre-weaning socialisation with non-littermates reduced the aggression of piglets after post-weaning regrouping. There was moderate evidence (in the six out of seven papers that measured skin lesions) that piglets with pre-weaning socialisation had fewer skin lesions post-weaning than non-socialised piglets. There was insufficient evidence (in one out of two papers that measured cortisol levels) supporting lower cortisol levels in socialised piglets post-weaning. The current evidence (in the four out of five papers that measured ADWG) did not find a significant improvement in ADWG with pre-weaning socialisation.

## Summary of the evidence

**Camerlink et al. (2018):** 

Population:	[Sow: Large White × Landrace] x [Boar: American Hampshire], Piglets, (UK).
Sample size:	Before weaning and before regrouping: 65 litters (683 piglets).After regrouping: 369 piglets (piglets below 12 kg, n = 11 piglets, were excluded). 10–13 piglets per group (average 12).
Intervention details:	Pre-weaning treatment groups: Treatment group was allocated based on parturition date. Group 1 control treatment (CON): n = 33 litters. Housing design: Littermate-only with sow in a farrowing pen until weaning. Group 2 socialisation treatment (SOC): n = 32 litters. Socialisation housing design: Littermate-only with sow in a farrowing crate until day 14, then day 14 socialisation facilitated by an opening on the barrier between neighbouring farrowing crates. Socialisation group size: two litters pen (socialisation group size is the number of litters that interact with one another during lactation).
Study design:	Non-randomised controlled trial.
Outcome studied:	Weaning age: day 26. Socialisation length: 12 days. Regroup condition: Piglets were regrouped at 8 weeks of age. Selected at least 2 piglets from each of three to four litters in the same pre-weaning treatment group to form post-weaning groups. Balanced group composition between treatments. Post-weaning group size: 10–13 piglets per group (average 12), from three to four litters. SOC = 18 groups. SOC piglets were not regrouped with previously socialised piglets. CON = 14 groups. Behavioural indicators: Skin lesion (in the morning before regrouping, 24 hours after regrouping, 3 weeks after regrouping).
Main Findings (relevant to PICO question)	Behavioural indicators: Skin lesion: 24 hours after regrouping. Total skin lesions: SOC < CON (significant, P = 0.045). Front: no significant difference. Middle: SOC < CON (significant, P = 0.047). Rear: SOC < CON (significant, P = 0.03). 3 weeks after regrouping (11 weeks of age). No significant difference between groups.
Limitations:	Only 369/683 (54%) piglets were regrouped and the selection criteria was not specified. Regrouping did not take place immediately at weaning. Skin lesion level before regrouping was not reported. Different group sizes after regrouping was not explained. The number of assessors for skin lesions was not specified. Exact number of piglets in each pre-weaning treatment group was not stated. The proportion of familiar piglet to unfamiliar piglet in a pen (familiarity ratio) after regrouping was not specified. Familiarity ratio is the number of familiar piglets (littermates or previously in same socialisation group) to the number of unfamiliar piglets (non-littermates and previously not in same socialisation group).

**D’Eath (2005):** 

Population:	[Sow: Large White X Landrace] x [Boar: Large White], Piglets, (UK).
Sample size:	Before weaning: 16 litters (198 piglets). Selected based on: close parturition time, with at least nine piglets (preferred).After weaning: 128 piglets. Selected eight healthy focal piglets (at least 6 kg) randomly from each litter, housed in one pen until regrouping.
Intervention details:	Pre-weaning treatment groups: Group 1 (control): no socialisation, n = 8 litters. Housing design: littermate-only with sow in a farrowing crate until weaning day 30. Group 2 (socialised): n = 8 litters. Socialisation housing design: littermate-only with sow in a farrowing crate until day 10, then socialise days 10–30 by removing a barrier between two adjacent pens. Socialisation group size: two litters per pen.
Study design:	Non-randomised controlled trial.
Outcome studied:	Weaning age: day 30. Socialisation length: 20 days. Regroup condition: Regrouped at day 50. Four piglets were selected at random from two unfamiliar litters. Post-weaning group size: eight piglets. Familiarity ratio: 1:1. Growth performance: Mean weight gain/Average daily weight gain (ADWG): days 0 (at birth), 9, 15, 21, 30, 45, 50, 55 and 60 of age. (Entire experiment and after regrouping). Behavioural indicators: Behavioural record. Fight (reciprocal). Bullying (one-sided). Skin lesions: days 51, 52 and 60. Food competition tests: days 37, 43 (pre-regrouping) and 57 (after regrouping).
Main Findings (relevant to PICO question)	Growth performance: Mean weight gain / Average daily weight gain (ADWG): no significant difference between treatment groups for the entire experiment or after regrouping. Behavioural indicators: Behavioural record. Day 50, latency to the first fight: socialised group < control group (significant, P = 0.034). Day 50, mean duration of fights: control group > socialised group (significant, P = 0.046). Day 51, frequency of bullying: control group > socialised group (significant, P = 0.039). Day 51, for fights with clear winner, the proportion of fights initiated by the eventual winner: socialised group > control group (significant, P = 0.048). Skin lesions: By day 61: socialised group < control group (significant, P = 0.008). Food-competition tests: result was not reported in D’Eath (2005) paper.
Limitations:	Treatment group allocation method was not stated. Number of piglets in each pre-weaning treatment group was not specified. Number of post-weaning groups from each pre-weaning treatment group was not specified. Total number of piglets was unknown after regrouping at day 50. The regrouping was not stated to be within the pre-weaning treatment group. While two assessors were used for skin lesions, due to significant systematic difference, the second person’s data was discarded, resulting in insufficient data at day 51 and reduced sample size at day 52, n = 96; day 60, n = 80. Regrouping did not immediately happen at weaning. Sampling frequency in a day or length of recording for aggressive behaviours were not specified. Food competition test result was not reported in D’Eath (2005) paper.

**Fels et al. (2021):** 

Population:	Breed unknown, piglets, (Germany).
Sample size:	Before weaning: 16 litters. After weaning: 90 piglets (10 piglets per group). Selection criteria unknown.
Intervention details:	Pre-weaning treatment groups: Group 1 Control (Co): no socialisation, n = 8 litters. Housing design: littermate-only with sow in a farrowing crate until weaning. Group 2 Group housing (Gr): socialisation, n = 8 litters. Socialisation housing design: five farrowing pens with a central common socialisation area which was accessible by the piglets at day 10 onwards. Socialisation group size: five litters per pen.
Study design:	Non-randomised controlled trial.
Outcome studied:	Weaning age: 35 days. Socialisation length: 25 days. Regroup condition: Gr/Gr: five Gr littermate + five Gr littermate, familiar piglets (Not examined in this review because there was no post-weaning mixing of unfamiliar piglets). Gr/Co: five Gr littermate + five Co littermate, unfamiliar piglets. Co/Co: five Co littermate + five Co littermate, unfamiliar piglets. All post-weaning groups were balanced by weight and sex. Post-weaning group size: 10 piglets per group. Familiarity ratio: Gr/Gr: N/A. Gr/Co: 1:1. Co/Co: 1:1. Behavioural indicators: Aggressive interactions (days 35–36) through video recording. Total number of aggressive interactions. Result of aggressive interactions. Total number of aggressive interactions between littermates (LM). Total number of aggressive interactions between non-littermates (non-LM). Skin lesions (before weaning, and day 39).
Main Findings (relevant to PICO question)	Behavioural indicators: Aggressive interactions (days 35–36): Total number of aggressive interactions: Gr/Gr < Gr/Co < Co/Co (trend only). No significant difference between Gr/Co and Co/Co. Total number of aggressive interactions with unclear results: Co/Co > Gr/Gr or Gr/Co (significant, P < 0.05). Total number of aggressive interactions between LM: Co/Co > Gr/Co or Gr/Gr (significant, P < 0.05). Total number of aggressive interactions between non-LM: Gr/Co and Co/Co did not differ. Within the Gr/Co group, total number of aggressive interactions, and number of aggressive interactions between LM: Gr > Co (trend only). Skin lesions (before weaning day 39): No significant difference between treatment groups before weaning. Less skin lesions: Gr/Gr < Gr/Co < Co/Co (significant, P < 0.05).
Limitations:	The sample size before weaning was not reported. Treatment group allocation method was not stated. The socialisation group (Gr) had more space allowance than the control group (Co) during lactation, which might be significant. While the total number of piglets for regrouping was specified, the number of groups in each regrouping condition (e.g., Gr/Gr) was not stated. The number of and the identity of the person who assessed the behaviours through videotape was not specified. Aggressive behaviours were not defined clearly. The skin lesion scoring system from 0–3 was vaguely defined as low to high grade.

**Ji et al. (2021):** 

Population:	Large White x DM (Duroc x Min Pig [YDM]), (China).
Sample size:	Before weaning: 12 litters (133 piglets, 11.2 ± 1.5 piglets per pen). After weaning: 12 litters (120 piglets), selected 10 piglets per litter (four litters in each treatment group), selected with similar body weight, sex balanced.
Intervention details:	Pre-weaning treatment groups: Piglets were randomly allocated into a treatment group. Group 1: control group, no contact or socialisation (CON), n = 4 litters (43 piglets). Housing design: littermate-only with sow in a farrowing crate until weaning. Group 2: intermittent contact / socialisation group (IM), n = 4 litters (46 piglets). Socialisation housing design: littermate-only with sow in a farrowing crate on days 1–14, then two neighbouring litters socialised in shared activity area on days 14–35 for 3 hours per day. Socialisation group size: two litters per pen (socialisation group size is the number of litters that interact with one another during lactation). Group 3: continuous contact / socialisation group (CM), n = 4 litters (44 piglets). Socialisation housing design: littermate-only with sow in a farrowing crate on days 1–14, then two neighbouring litters socialised in shared activity area on days 14–35 with 24 hours access. Socialisation group size: two litters per pen.
Study design:	Randomised control trial.
Outcome studied:	Weaning age: 35 days. Socialisation length: 21 days. Regroup condition: CON: Randomly mixed according to weight and sex, unfamiliar piglets. IM: Mixed with non-adjacent litters in same treatment group, unfamiliar piglets. CM: Mixed with non-adjacent litters in same treatment group, unfamiliar piglets. Post-weaning group size: 10 piglets per group / pen. Physiological indicators: Blood, day 42 (1 week after mixing). Interleukin-1β (IL1β). Cortisol (COR). Interleukin-6 (IL6). Interleukin-10 (IL10). Brain derived neurotrophic factor (BDNF). Growth performance: Day 63. Group uniformity: coefficient of variation (CV). Feed to meat ratio. Average daily weight gain (ADWG). Behavioural indicators: Days 35–36, and days 35–38, 30-second scan sampling every 10 minutes. Aggressive behaviours: fighting, head knocks. Social interactions: oral-nasal contact, mounting, no reaction, avoiding, return approach.
Main Findings (relevant to PICO question)	Physiological indicators: IL1β: CM > CON > IM (significant, P < 0.001). COR: IM significantly lower than CON and CM (P = 0.005). IM < CON < CM (trend only). BDNF: IM > CON (significant, P = 0.024). Growth performance: From weaning until the end of nursery (day 63). CV: IM significantly lower CV than CM and CON (P = 0.043). Feed to meat ratio: CM significantly higher than IM and CON (P = 0.001). Average daily weight gain (ADWG): IM > CM (significant, P = 0.005)**, **but no significant difference between CON and IM / CM. Behavioural indicators: Aggressive behaviours: Fighting: CON > IM > CM (significant on days 35–36, P = 0.001; days 35– 38, P < 0.001). Head knocks: control group significantly more than socialised group (days 35–36, P = 0.003; days 35–38, P = 0.001). Social interactions: Oral-nasal contact: CON significantly more than socialised group (days 35–36, P < 0.001), CON > IM > CM (significant on days 35–38, P < 0.001). Mounting: no significant difference (days 35–36), IM significantly lower than the other groups (days 35–38, P = 0.036). No reaction: CM significantly lower than the other groups (days 35–36, P = 0.018; days 35–38, P < 0.001). Avoiding: CM significantly higher than the other groups (days 35–38, P = 0.008). Return approach: CM significantly higher than the other groups (days 35–36, P = 0.001; days 35–38, P < 0.001).
Limitations:	The mechanism of randomisation for treatment group allocation was not reported. The study was not blinded. The socialisation group (IM and CM) had more space allowance than the control group (CON) during lactation, which might be significant. The proportion of familiar piglet to unfamiliar piglet (familiarity ratio) in a pen after regrouping was not specified. There was no baseline for the physiological indicators established prior to regrouping. Behavioural observations were recorded by one observer only.

**Kanaan et al. (2012):** 

Population:	York X Landrace, Piglet, (US).
Sample size:	56 litters. Before weaning: 48 litters. After weaning: 192 piglets selected (48 post-weaning groups of piglets consisting of 16 post-weaning groups per pre-weaning treatment group).
Intervention details:	Pre-weaning treatment groups: Group 1 control, no socialisation (CM0): n = 16 litters. Housing design: Standard farrowing crates from birth until weaning on day 18. Group 2 socialisation with one unfamiliar litter (CM1): n = 16 litters. Socialisation housing design: co-mingle with one unfamiliar litter from days 10–18. Socialisation was facilitated by removing the barrier between adjacent farrowing pens. Socialisation group size: two litters per pen (socialisation group size is the number of litters that interact with one another during lactation). Group 3 socialisation with two unfamiliar litter (CM2): n = 16 litters. Socialisation housing design: co-mingle with one unfamiliar litter from days 10–14 then another unfamiliar litter from days 14–18. Socialisation was facilitated by removing the barrier between adjacent farrowing pens. Socialisation group size: two litters per pen.
Study design:	Non-randomised controlled trial.
Outcome studied:	Weaning age: approximately day 18. Socialisation length: 8 days. Regroup condition: One male and one female from one litter, plus one male and one female from another litter, within the same pre-weaning treatment group. Both pairs of littermates had similar weight and were unfamiliar to one another. Post-weaning group size: four piglets. Familiarity ratio: 1:1. Growth performance: Mean daily weight gain / average daily weight gain (ADWG): days 2, 9, 13, 17, 21 and 25. Behavioural indicators: Behavioural tests. Behaviour: 48 hour post-mixing (using 10 min scan sampling). Ear injuries: days 2, 9, 13, 17, 21 and 25. Average ear injuries score for the entire experiment and time points. Social challenge: days 13, 17 and 21. Two unfamiliar piglets = one focal piglet + one piglet from another litter. Observed social interactions for 10 min. Social recognition: days 16 and 25. Social investigation (frequency, duration, and latency).
Main Findings (relevant to PICO question)	Growth performance: Mean daily weight gain / Average daily weight gain (ADWG): no significant difference found for the entire experiment or at any time point. Behavioural indicators: Ear injuries: no significant difference found. Behaviour: 48 hours post-mixing. Belly-nosing: CM2 spent a higher proportion of observations performing belly-nosing than CM0 (significant, P = 0.05). CM2 > CM1 > CM0 (trend only). All aggressive interactions: CM1 spent a lower proportion of observations in this than CM0 (significant, P < 0.05). CM1 < CM2 < CM0 (trend only). Aggression towards non-littermates: CM1 spent a lower proportion of observations in this than CM0 (significant, P < 0.05). CM1 < CM2 < CM0 (trend only). All activities with non-littermates: CM1 < CM0 (trend only), CM2 no significant difference from the two. Behavioural tests – not examined in this review because it did not specifically report post-weaning results.
Limitations:	Treatment group allocation method was not stated. The pre-weaning selection criteria of 48 litters out of 56 litters was not explained. Outcomes were measured in focal piglets only. Selection criteria for focal piglets included sex and weight only. Ear injury scoring system was not quantified (vague definition such as score 1 = few scratches). For social challenge or social recognition, the post weaning result was not isolated from the pre weaning result. Total number of piglets before weaning was unknown. Number of piglets in each treatment group pre-weaning and post-weaning was not specified. The number of assessors for behaviours and ear injuries was not specified.

**Salazar et al. (2018):** 

Population:	Breed unknown, piglet, (Spain).
Sample size:	Before weaning: 52 litters. After weaning: 16 weaning pens, about 39 piglets per pen (range from 31–45).
Intervention details:	Pre-weaning treatment groups: Piglets were randomly allocated into a treatment group. Group 1 no socialisation / control (CON): n = 12 litters. Housing design: littermate-only with sow in a farrowing pen until weaning. Group 2 socialisation from day 7 postpartum to day 25 (M7): n = 20 litters (262 piglets). Socialisation housing design: littermate-only with sow in a farrowing pen until day 7, then the barrier between two adjacent farrowing pens was removed at day 7 post-partum. Socialisation group size: two litters per pen (socialisation group size is the number of litters that interact with one another during lactation). Group 3 socialisation from day 14 postpartum to day 25 (M14): n = 20 litters (253 piglets). Socialisation housing design: littermate-only with sow in a farrowing pen until day 14, then the barrier between two adjacent farrowing pens was removed at day 14 post-partum. Socialisation group size: two litters per pen.
Study design:	Randomised control trial.
Outcome studied:	Weaning age: 25 days. Socialisation length: 18 days for M7, 11 days for M14. Regroup condition: Regroup with unfamiliar piglets from the same treatment group based on similar body size. For outcome measurement: Six piglets per litter of the 12 largest litters in each pre-weaning treatment group were selected (heaviest male and female, lightest male and female, median pen weight male and female). In total, 72 piglets were selected from each pre-weaning treatment group for post-weaning outcome measurement. Physiological indicators: Salivary cortisol. 1 day before weaning (premixing), 1 day after weaning (+1), 2 days after weaning (+2) in a sample of 72 piglets per treatment group. Basal level of salivary cortisol = premixing / preweaning level. Growth performance: Average daily weight gain (ADWG). Weighed at days 1, 7, 14, 21, 28 and 35 after birth. Behavioural indicator: Number of skin lesions (two observers with interobserver reliability of r = 0.7). Before weaning: 1 day before co-mingling (premixing, –1), 1 day after co-mingling (+1), 2 days after co-mingling (+2) for all piglets. At weaning: 1 day before weaning (premixing, –1), 1 day after weaning (+1), 2 days after weaning (+2) in a sample of 72 piglets per treatment group.
Main Findings (relevant to PICO question)	Physiological indicators: Salivary cortisol. No significant difference between basal levels (pre-mixing) of cortisol among treatment groups. CON piglets showed greater increase in cortisol than M7 and M14 piglets (significant, P = 0.02 respectively). Impact of sampling time: later sampled piglets showing significantly higher cortisol level (P = 0.01). Growth performance: ADWG Week 1 (lactation): CON > M7 (significant, P = 0.01); CON > M14 > M7 (trend). Week 2 (M7 socialisation starts): no significant difference found. Week 3 (M14 socialisation starts): CON > M7 (significant, P < 0.01); CON < M14 (significant, P = 0.01). Week 4 (at weaning): all group dropped, but no significant difference among treatments. Week 5 (1 week after weaning): all slightly increased, but no significant difference among treatments. Behavioural indicators: Number of skin lesions. Compare pre-weaning vs post-weaning: M14, no difference. CON, significantly higher post-weaning lesions (+1 or +2) than pre-weaning (-1) (P < 0.001). M7, significantly lower post-weaning lesions (+1 or +2) than pre-weaning (–1) (P = 0.007). However, number of skin lesions on any day (–1, +1, +2) do not significantly differ among treatment groups.
Limitations:	Total numbers of piglets before and after weaning were not specified. Number of piglets in pre-weaning control group was unknown. The mechanism of randomisation for treatment group allocation was not reported. Post-weaning group size was unknown. Only selected piglets were measured for skin lesions and salivary cortisol. Though measurement was conducted in a sample of 72 piglets per treatment group, the final result presented for skin lesion contained less data (only CON n = 66, M7 n = 60, M14 n = 60). No explanation was given for the missing data. The proportion of familiar piglet to unfamiliar piglet in a pen (familiarity ratio) after regrouping was not specified.

**Schrey et al. (2019):** 

Population:	Breed unknown, piglets, (Germany).
Sample size:	Before weaning: 34 litters (400 piglets). After weaning: 14 litters (70 piglets).
Intervention details:	Pre-weaning treatment groups: Group 1 conventional individual housing / control / no socialisation (IH): n = 10 litters (126 piglets). Housing design: littermate-only with sow in a farrowing pen until weaning. Group 2 group housing / socialisation (GH): n = 24 litters (274 piglets). Socialisation housing design: five farrowing pens with a central common area which was made accessible after the first piglet can pass through the flexible steps. Socialisation group size: five litters per pen (socialisation group size is the number of litters that interact with one another during lactation).
Study design:	Non-randomised controlled trial.
Outcome studied:	Weaning age: day 35. Regroup condition: IH: n = six litters (30 piglets). GH: n = eight litters (40 piglets). Five piglets from two unfamiliar litters in the same pre-weaning treatment groups. Balanced by weight and sex. Post-weaning group size: 10 piglets per group. Familiarity ratio: 1:1. Growth performance: Average daily weight gain (ADWG) (days 35, 39 and 63). Behavioural indicators: Skin lesion (before weaning vs day 39).
Main Findings (relevant to PICO question)	Growth performance: ADWG: higher daily weight gain in GH than IH (days 35–39, P = 0.003; days 35–63, P = 0.085). Behavioural indicators: Skin lesion. IH: Skin lesion significantly increased (days 35–39, P = 0.014). GH: No significant increase (days 35–39).
Limitations:	Treatment group allocation method was not stated. The GH had more space allowance than the IH during lactation, which might be significant. The start day of socialisation during lactation was not specified, and based on its description, the start day may have varied in different litters. Socialisation length was unknown. The selection criteria for piglets to proceed to regrouping was not stated (sudden reduction from 400 to 70 piglets). Skin lesions were assessed by one trained observer only.

**Verdon et al. (2016):** 

Population:	Landrace x Large White, piglets, (Australia).
Sample size:	Before weaning: 72 litters (642 piglets). For group lactation pens, n = 6 sows and litters per pen. After weaning: The average group size at mixing was 35.7 piglets (range 29–40).
Intervention details:	Pre-weaning treatment groups: Initially, sows were allocated to either farrowing crate (FC, n = 36 sows) or Piglet and Sow Alternative Farrowing Environment (PigSAFE) (PS, n = 36 sows). At day 14, 12 sows in FC and 12 sows in PS were randomly selected to group lactation pens (GL-FC and GL-PS) respectively. Group 1 farrowing crate, no socialisation (FC): n = 24 litters. Housing design: In a farrowing crate with sow and littermate-only from days 0–27. Group 2 PigSAFE pen, no socialisation (PS): n = 24 sows / litters, number of piglets unknown. Housing design: In a PigSAFE pen with sow and littermate-only from days 0–27. Group 3 farrowing crate then group lactation, with socialisation (GL-FC): n = 12 litters, six sows/litters per group lactation pen. Socialisation housing design: In a farrowing crate with sow and littermates from days 0–14, then transferred to group lactation and stay until weaning days 27. Socialisation group size: six litters per pen (socialisation group size is the number of litters that interact with one another during lactation). Group 4 PigSAFE pen then group lactation, with socialisation (GL-PS): n = 12 litters, six sows / litters per group lactation pen. Socialisation housing design: In a PigSAFE pen with sow and littermates from days 0–14, then transferred to group lactation and stay until weaning day 27. Socialisation group size: six litters per pen.
Study design:	Non-randomised controlled trial.
Outcome studied:	Weaning age: average 27.3 days (range 24–30). Socialisation length: 13 days. Regroup condition: FC = four litters, unfamiliar. PS = four litters, unfamiliar. GL = two litters from GL-FC and 2 litters from GL-PS, unfamiliar, such to maximise the number of unfamiliar group lactation litters being mixed post-weaning. Post-weaning group size: average 35.7 piglets (range 29–40). Familiarity ratio / unfamiliar piglets percentage: GL: 50% (range 44–56%), FC: 75% (range 70–81%), PS: 75% (range 63–86%). Behavioural indicators: Behaviours: for 2 hours after regrouping: Bouts of aggression. Fights (latency to fight, fighting frequency, average fight duration and total duration fighting). Skin lesions (six piglets per litter randomly selected, n = 432 piglets): on the day before weaning (day 26), and 24 hours post-mixing (day 28). Measured total number of skin lesions on three zones (front, middle and rear).
Main Findings (relevant to PICO question)	Behavioural indicators: Behaviours: for 2 hours after regrouping. Bouts of aggression: GL < FC or PS (significant, P < 0.01). Latency to fight: no significant difference. Fighting frequency: GL < FC or PS (significant, P < 0.01). Average fighting duration: GL < FC (significant, P = 0.04) but not PS. Total duration fighting: GL < FC (significant, P < 0.01), but not PS. Skin lesions: GL < FC or PS 24 hours post-mixing (significant, P < 0.01).
Limitations:	While GL-FC and GL-PS were randomly selected from FC and PS, the allocation method of sows / litters to PS and FC was unknown. There was a range of weaning age. GL piglets had a higher proportion of familiar piglets in the pens after regrouping. Skin lesions were assessed in randomly selected focal piglets rather than all piglets. The number of assessors for behaviours and skin lesions was not specified. Number of piglets in each pre-weaning treatment group, and total number of piglets after weaning were unknown.

**Verdon et al. (2019):** 

Population:	Large White × Landrace, piglets, (Australia).
Sample size:	Before weaning: 36 litters (378 piglets). After weaning: 30 litters (198 piglets). 10–14 piglets per pen, five pens per treatment. Selection of piglets was to ensure the balance of the two litters in terms of: Average piglet weight at weaning in each litter CV (the term was not expanded in original study) in piglet weight at weaning. Litter age. Sex.
Intervention details:	Pre-weaning treatment groups: Treatment groups were balanced for sow’s parity, sow weight and litter size. Group 1 farrowing crate for the whole lactation period (no socialisation / control) (FC): n = 12 litters. Housing design: sows and litters remained in individual farrowing crates for the whole lactation period. Group 2 farrowing crate and then transferred to group lactation (GL) pens at day 7 post-partum (socialisation from day 7) (GL7): n= 12 litters. Socialisation housing design: at average day 7 post-partum, 12 sows and their litters were transferred into either: Five sows / litters group lactation pen. Seven sows / litters group lactation pen. Socialisation group size: five litters or seven litters per pen (socialisation group size is the number of litters that interact with one another during lactation). Group 3 farrowing crate and then transferred to group lactation pens at day 14 post-partum (socialisation from day 14) (GL14): n = 12 litters. Socialisation housing design: At average day 14 post-partum, 12 sows and litters were transferred into either: Five sows / litters group lactation pen. Seven sows / litters group lactation pen. Socialisation group size: five litters or seven litters per pen.
Study design:	Non-randomised controlled trial.
Outcome studied:	Weaning age: average 26.7 days (range from 22–29 days). Socialisation length: 13 days. Regroup condition: Equal proportions from two unfamiliar litters (5–7 piglets from each litter), from the same treatment groups (refer to sample size section for selection criteria). Post-weaning group size: 10–14 piglets. The group size was dependent on: Healthy piglets available per litter. Number of litters available in the smaller group lactation pen (the five litter pen). Group size for each condition: FC: five pens of 14 piglets. GL7: four pens of 14 piglets and one pen of 10 piglets. GL14: two pens of 14, two pens of 12, and one pen of 10 piglets. Familiarity ratio: 1:1. Behavioural indicators: Behavioural recording for 3.5 hours (30 minute interval) after post-weaning mixing. Aggression: frequency of aggressive bouts, frequency and duration (total, average) of fights and bullying events. Dyadic familiarity and aggression: whether each interaction involves a familiar piglet or non-familiar piglet. (The outcome was not relevant to PICO question and not examined in this review).
Main Findings (relevant to PICO question)	Behavioural indicators: Behavioural recording for 3.5 hours (30 min interval) after post-weaning mixing. Aggression Frequency of aggressive bouts:** **FC > GL7 (trend only), FC > GL14 (trend only). Frequency of fights: FC > GL7 (significant, P < 0.001), FC > GL14 (significant, P < 0.001). Duration of fights: FC > GL7 (significant, P < 0.001), FC > GL14 (significant, P < 0.001). Frequency of bullying: FC > GL7 (trend only), FC > GL14 (trend only). Aggressive bouts X Time: aggressive bouts delivered by all piglets declined over time, but GL7 and GL14 piglets reached baseline quicker than FC piglets (trend only). During 30–60 minutes post mixing, FC piglets spend more time fighting than GL7 and GL14 (significant, P < 0.05); but no difference among groups found in other time periods.
Limitations:	The socialisation group (GL7 and GL7) had more space allowance than the control group (FC) during lactation, which might be significant. The exact number of piglets in each pre-weaning treatment group was not specified. Group lactation pens for socialisation were not identical in structure: one accommodated five sows and litters while the other accommodated seven sows and litters. This might affect the level of socialisation. The method for allocation of the sows and litters to either five or seven litter group lactation pens was unclear. There was a range of weaning age. Group size after regrouping was not uniform (10–14 piglets), resulting in different space allowance per piglet. The number of observers, and skill level of the observer for behavioural recording were not specified. The behavioural outcomes were measured for only 3.5 hours. The baseline for ‘Aggressive bouts X Time’ was compared to but the establishment of such baseline was not specified.

## Appraisal, application and reflection

Comparison across all papers was challenging due to highly variable study designs including sample size, socialisation housing design, socialisation length, weaning age, outcome studied, and method of sample collection (sample type, date, time). Unclear reporting in all papers jeopardised fair comparisons, such as unspecified piglet numbers in each pre-weaning treatment group and unexplained reduction in the number of piglets from pre-weaning to post-weaning regrouping. The method for treatment group allocation was not detailed in seven out of nine papers, which included the two randomised control trial papers. Most importantly, the familiarity ratio was not specified by Ji et al. (2021), Salazar et al. (2018), and Camerlink et al. (2018), which determined the level of social challenge that a piglet would face post-weaning at regrouping, and which ideally should be constant across all post-weaning groups.

There were other aspects of study design that undermined the strength of evidence in individual papers such as that of Verdon et al. (2019) and Fels et al. (2021). Verdon et al. (2019) did not have a consistent socialisation housing design for all piglets within one socialisation treatment group: for instance, in treatment group GL7, piglets were socialised in either a five-litter pen or seven-litter pen, resulting in different socialisation group size, and thus different levels of socialisation within the treatment group.

Ideally, piglets should be regrouped within the same treatment groups (i.e., socialised piglets with unfamiliar socialised piglets only) such that post-weaning groups directly represent the original treatment groups, but Fels et al. (2021) mixed socialised piglets with non-socialised piglets (Gr/Co) in post-weaning regrouping and compared these to non-socialised post-weaning groups (Co/Co). As a result, the magnitude of the difference between Gr/Co and Co/Co would be less than that between pure socialised post-weaning groups and pure non-socialised post-weaning groups.

### Physiological indicator – Cortisol

Salazar et al. (2018) found that socialised piglets had a statistically significant, smaller increase in cortisol from pre-weaning to post-weaning than non-socialised piglets (P = 0.02), but Ji et al. (2021) found a higher, though not statistically significant, cortisol level in socialised piglets (CM) than non-socialised piglets (CON) post-weaning. In this section of discussion, only results from treatment groups with continuous socialisation (i.e., M7 and M14 in Salazar et al., 2018; and CM in Ji et al., 2021), in which piglets had 24-hour full access to non-littermates, were included as that of socialised piglets due to similar level of socialisation.

This disagreement in results could be a function of the difference in study design, whereby the change in cortisol level (pre-weaning to post-weaning) between treatment groups was compared by Salazar et al. (2018) but not in the paper of Ji et al. (2021), where basal cortisol pre-weaning was not measured. There is a possibility that the higher post-weaning cortisol level in socialised piglets measured by Ji et al. (2021) reflected a higher pre-weaning cortisol level among the piglets in the socialisation treatment group than piglets from the non-socialisation treatment group due to additional social stress, different housing designs, and more frequent handling and monitoring by humans to facilitate socialisation arrangements starting at Day 14, such that a potentially smaller increase in cortisol level for the socialised piglets on post-weaning regrouping compared to the non-socialised piglets was not captured. In addition, sample collection at different times of the day might affect the result, because piglets have a circadian rhythm for cortisol levels (Gallagher et al., 2002) and piglets sampled in a later time showed significantly higher cortisol levels (Salazar et al., 2018). Other factors to consider could be the unspecified familiarity ratio in both papers and different sampling dates.

It is interesting to note that Ji et al. (2021) collected blood samples while Salazar et al. (2018) collected salivary samples. Both sample types are validated for cortisol measurement and this difference will have had minimal effect on the result (Mormède et al., 2007).

### Behavioural indicators – Aggression

The main findings in all six papers supported that piglets with pre-weaning socialisation were less aggressive than piglets without pre-weaning socialisation when regrouped post-weaning. This was reflected across various outcomes such as less fighting, shorter fighting duration, and more fights with clear results in socialised piglets post-weaning.

Comparison between papers was extremely challenging. Besides the above-mentioned differences in study design, other issues include unclear behavioural sampling interval (D’Eath, 2005; Fels et al., 2021; and Verdon et al., 2016) and an unspecified number of observers and unknown inter-observer reliability (all papers except Ji et al., 2021). The variation in post-weaning group size across all papers and within papers (Verdon et al., 2016; and Verdon et al., 2019) could also affect the result, as the level of aggression would be higher in a smaller group (Andersen et al., 2004). Although, it should be noted that the variable post-weaning group size in Verdon et al. (2019) could be an acceptable study design as it achieved a uniform familiarity ratio and thus an equal chance of contact with unfamiliar piglets in all post-weaning groups.

Another important feature limiting the comparison was that aggression was measured by a set of behaviours, which was different in all papers except that of Verdon et al. (2016) and Verdon et al. (2019). Even when the same behavioural term was used, such as ‘fight’ by Ji et al. (2021) and Verdon et al. (2016), their descriptions were different in terms of action and duration. Based on current research, there is no existing standardised set of behaviours that are typical of aggression in post-weaning piglets.

### Behavioural indicators – Skin lesions

Six out of seven papers, except Kanaan et al. (2012), found that pre-weaning socialisation significantly reduced the amount and the severity of skin lesions during post-weaning regrouping. This could be shown by either a smaller increase from pre-weaning to post-weaning level within socialised piglets compared to the increase seen within the control, or more skin lesions in the control than the socialised piglets post-weaning. This result is expected because usually skin lesions are inflicted during fights and thus are related to aggression (Stukenborg et al., 2011) of which piglets with pre-weaning socialisation showed a lower level on post-weaning regrouping as discussed above.

Several factors that might contribute to the non-significant result in Kanaan et al. (2012), including that it had the shortest socialisation length of all papers (8 days vs. 11–25 days), the smallest post-weaning group size of all papers (4 piglets vs. 8–40 piglets), and the measurement of skin lesions was limited to lesions on the ears only. While other papers measured three or more body parts, the comparison across papers was still difficult because the body parts scored might include variable combinations of head, ear, shoulder, neck, tail, and so on.

One more feature that made comparison difficult was that skin lesions were recorded by two methods, either the total number of skin lesions (Camerlink et al., 2018; D’Eath, 2005; Salazar et al., 2018; and Verdon et al., 2016) or a cumulative skin lesion scoring index (Fels et al., 2021; Kanaan et al., 2012; and Schrey et al., 2019). In terms of the latter, Schrey et al. (2019) provided clear definitions consisting of both the number of lesions and severity of the lesion, while Fels et al. (2021) and Kanaan et al. (2012) had vague and less objective definitions for each score, such as ‘Score 1 = few scratches’ (Kanaan et al., 2012).

It is interesting to note that the effect of pre-weaning socialisation on skin lesions may be short-lived because Camerlink et al. (2018) found no significant difference between treatment groups at three weeks after post-weaning regrouping. The lack of long-term significant results could be due to the decline in fights in all post-weaning groups after the re-establishment of a new social hierarchy in the group within 48 hours (Meese & Ewbank, 1973; and Tong et al., 2019), and could be due to the healing of existing partial-thickness wounds, which could re-epithelialise within 5 days (Singer & McClain, 2003), while closure of full-thickness wounds would take longer than 3 weeks (De Coninck et al., 1996).

### Growth performance indicator – Average daily weight gain (ADWG)

Four out of five papers did not find a statistically significant difference in ADWG between socialised piglets and non-socialised piglets post-weaning (D’Eath, 2005; Ji et al., 2021; Kanaan et al., 2012; and Salazar et al., 2018).

The feature in Schrey et al. (2019) that possibly contributed to a significantly higher ADWG in socialised piglets than non-socialised piglets in the first four days post-weaning (P = 0.003) was group housing to facilitate socialisation during lactation. A similar result was obtained by Kutzer et al. (2009): although post-weaning regrouping with unfamiliar piglets in the socialisation treatment group was not performed in this paper, piglets in group housing systems had a significantly higher ADWG post-weaning than piglets in individual farrowing crates. It is likely that a significantly higher ADWG can be observed among socialised piglets if they experience significantly less social stress when they move from pre-weaning group housing to post-weaning groups. Specifically, socialised piglets in Schrey et al. (2019) might have experienced a significant downgrade of the social challenge from four litters of unfamiliar non-littermates in pre-weaning group housing to only five unfamiliar piglets in post-weaning groups; while in other papers, the transition from pre-weaning socialisation group size of two litters to post-weaning group size of 10 piglets did not have significant impact on piglets’ social stress and thus not have significant changes in ADWG. This hypothesis can be tested in future studies by measuring the changes in cortisol levels from pre-weaning to post-weaning to demonstrate the significant reduction in social stress due to sharp decrease in group size.

### Application

Current evidence does not support a significant impact of pre-weaning socialisation on post-weaning weight gain, which may be a major setback for the adoption of socialisation strategy in commercial piggeries. However, since all papers did not find a negative impact of pre-weaning socialisation on weight gain, and most of the papers did find benefits such as reduced aggression and fewer skin lesions, pre-weaning socialisation can potentially improve the overall welfare of the piglets, which is also strongly demanded by consumers (Thorslund et al., 2017). Pre-weaning socialisation also has the potential to reduce the spread of greasy pig disease, because less fighting and fewer skin lesions means less opportunities for pathogenic strains of *Staphylococcus hyicus* to gain entry via skin wounds (Foster, 2012). Socialised piglets experiencing lower post-weaning stress may also have less disruption to intestinal morphology and barrier function, and thus potentially reduce their vulnerability to bacteria and endotoxins, and lower the incidence of diarrhoea (Tang et al., 2022).

Producers may be more willing to adopt pre-weaning socialisation strategies if the benefits of implementation can be proven to significantly outweigh the costs. To support this, further research must be run in commercial piggeries, to allow producers to make a comprehensive cost-benefit analysis. Benefits to be considered include increased feed conversion efficiency and thus lower cost of feed per animal (Ji et al., 2021), attracting a premium price with improved animal welfare (Thorslund et al., 2017), potential increase in ADWG (Schrey et al., 2019), more uniform group at selling (Ji et al., 2021) and so on. To demonstrate the profitability of pre-weaning socialisation practice, these benefits should significantly outweigh the costs involved with setting up new facilities for socialisation and management of the flow of socialised piglets. The benefit of anticipated improvement in consumer perception should also be considered, along with costs associated with marketing and building a positive brand image in relation to pre-weaning socialisation.

### Reflection

Future studies should report key features clearly, especially: sample size (number of piglets and litters before and after weaning, any exclusion or selection criteria), allocation method for treatment group, socialisation length, socialisation housing design, weaning age, whether post-weaning groups align with original treatment groups (i.e. regroup socialised piglets with unfamiliar socialised piglets, not socialised piglets with non-socialised piglets), group size after regrouping, familiarity ratio and methods of outcome measurement.

For outcome measurements, a diversity of physiological indicators can be considered, such as interleukins and brain-derived neurotrophic factor (BDNF) (Ji et al., 2021). Behavioural tests such as social recognition and social challenge can be used to determine the social skill of individual piglets (Kanaan et al., 2012), though they may be logistically more challenging to conduct.

For all indicators, establishing the basal level in each treatment group before weaning and measurement of change from pre-weaning to post-weaning will provide more accurate and more meaningful data on change in stress level and potentially yield more significant results than a mere comparison between treatment groups at each time point.

It is worth highlighting that a uniform familiarity ratio in all post-weaning groups, including either socialised or non-socialised piglets, is essential to ensure that an equal social challenge is faced by every piglet post-weaning. Post-weaning group size should also be kept constant because group size affects the level of agonistic behaviours (Andersen et al., 2004) being higher in smaller size groups, which potentially reflects its impact on the level of social challenge.

For behavioural-related indicators, the number of observers or assessors, the expertise of the observers or assessors, the scoring system or ethogram, and inter-observer reliability, should be well-defined and clearly described in the study. It is recommended to measure behaviours for at least 48 hours because this is the period with the most piglet activities relating to hierarchy establishment (Meese & Ewbank, 1973; and Tong et al., 2019). It will be very helpful if a list of behaviours that are more representative of aggression in weaners can be validated and established by future studies.

## Methodology

**Search strategy:** 

Databases searched and dates covered:	CABI: CAB Abstracts via Web of Science (1910–Jan 2023) BIOSIS Previews via Web of Science (1926–Jan 2023)
Search strategy:	CAB Abstracts: LA=(English) AND (TI=(pig OR piglet OR swine OR "sus scrofa")) AND (TS=(social* OR co-mingl*)) AND (TS=(wean* OR "early life" OR early OR "suckling pig" OR "baby pig" OR preweaning OR pre-weaning)) NOT (TS=(outdoor OR free-range)) BIOSIS Previews: LA=(English) AND (TI=(pig OR piglet OR swine OR "sus scrofa")) AND (TS=(social* OR co-mingl*)) AND (TS=(wean* OR "early life" OR early OR "suckling pig" OR "baby pig" OR preweaning OR pre-weaning)) NOT (TS=(outdoor OR free-range))
Dates searches performed:	10 Jan 2023

**Exclusion / inclusion criteria:** 

Exclusion:	Not primary research. Not in English. Not relevant to PICO question: Study of other species such as guinea pigs. Outdoor housing systems. Early socialisation is not the sole intervention in treatment group (for instance: if the treatment group has two types of enrichment implementing at the same time, toys + socialisation, the study will be excluded). Piglets were not regrouped with unfamiliar piglets after weaning. No behavioural, physiological or growth performance outcome of weaning stress was measured post-weaning. Not controlled trial studies. Not accessible by online databases (University of Sydney institutional access).
Inclusion:	Primary research. In English. Relevant to PICO question: Species, pigs. Life stages: Piglets pre-weaning to post-weaning. Indoor housing systems. Comparison of pre-weaning socialisation with non-littermates vs no pre-weaning socialisation with non-littermates. Socialisation is the sole enrichment condition in the treatment group. Measured a behavioural and/or physiological and / or growth performance outcomes relevant to weaning stress when regrouped with unfamiliar piglets post-weaning. Peer-reviewed. Controlled trial studies. Accessible by online databases (University of Sydney institutional access).

**Search outcome:** 

Database	Number of results	Excluded – Not primary study	Excluded – Not in English	Excluded – Not relevant to PICO	Excluded – Not controlled trial studies	Excluded –Not accessible by database	Total relevant papers
CAB Abstracts	473	53	60	351	0	0	9
Biosis Previews	348	43	0	299	0	0	6
Total relevant papers when duplicates removed							9

### ORCiD

Dongyue Du: https://orcid.org/0000-0003-3049-8535 Jenny-Ann Toribio: https://orcid.org/0000-0003-4262-2599

### Conflict of interest

The author declares no conflicts of interest.

## Disclaimer

Knowledge Summaries are a peer-reviewed article type which aims to answer a clinical question based on the best available current evidence. It does not override the responsibility of the practitioner. Informed decisions should be made by considering such factors as individual clinical expertise and judgement along with patient’s circumstances and owners’ values. Knowledge Summaries are a resource to help inform and any opinions expressed within the Knowledge Summaries are the author’s own and do not necessarily reflect the view of the RCVS Knowledge. Authors are responsible for the accuracy of the content. While the Editor and Publisher believe that all content herein are in accord with current recommendations and practice at the time of publication, they accept no legal responsibility for any errors or omissions, and make no warranty, express or implied, with respect to material contained within. For further information please refer to our Terms of Use.
